# Clinical and epidemiological profile of patients with mental disorders in a specialized outpatient clinic and its role in the psychosocial care network

**DOI:** 10.3389/fpsyt.2024.1274192

**Published:** 2024-01-24

**Authors:** Gláucio Silva Camargos, Mateus Afrânio Von Ancken Garcia, Carolina Andreazza de Almeida, Angélica Marta Lopes, Fábio Aparecido Borghi, Gerardo Maria de Araújo Filho, Luíz Carlos de Mattos, Cinara Cássia Brandão

**Affiliations:** ^1^Faculdade de Medicina de São José do Rio Preto – FAMERP, São José do Rio Preto, São Paulo, Brazil; ^2^Hospital de Base de São José do Rio Preto, São Paulo, Brazil

**Keywords:** psychosocial care network (PCN), mental health disorders, specialized outpatient clinic, generalized anxiety (GAD), recurrent depresive disorder

## Abstract

**Introduction:**

Mental health disorders (MHDs) are responsible for much impairment of quality of life in Brazil and worldwide. Early diagnosis and effective treatment strategies are required due to the heterogeneous symptoms and multifactorial etiology.

**Methods:**

A descriptive retrospective observational study was performed aiming to characterize the clinical and psychiatric profiles of patients with MHD attending a Brazilian public tertiary psychiatric outpatient clinic, which is a reference health service for more than 2 million inhabitants. Predominant clinical and sociodemographic aspects of patients were evaluated between March 2019 and March 2021.

**Results:**

A total of 8,384 appointments were analyzed. The majority of patients were female, and the mean age was 45 years old. Generalized anxiety disorder (GAD) was the most common MHD. The prevailing symptoms were sadness, anxiety, and irritability, with the most prescribed medications being selective serotonin reuptake inhibitors.

**Conclusion:**

The epidemiological characterization of mental disorders in specialized mental health outpatient clinics provides evidence for the establishment of more specific protocols and advocates a dimensional transdiagnostic approach as an aid to public mental health services.

## 1 Introduction

Mental health disorders are responsible for a significant amount of the impairment of quality of life and work incapacity in Brazil and around the world (1). These disorders include different clinical entities with varied symptoms but are mainly characterized by changes in the psychic, cognitive, and mood dimensions. Their multifactorial etiology involves psychological, genetic, social, and biological factors (2). With a significant impact on public health, among the most frequently reported complaints are depressive disorders, mood disorders (bipolar affective disorder—BAD), generalized anxiety disorder (GAD), and schizophrenia and other psychoses (3, 4). For studies on this topic, the concepts of dysfunction, disorder, and disease, respectively, complement the taxonomy of mental, psychic, and psychiatric concepts and are used to describe deviations that cause instability, pain, or impairment of these components (5, 6). These conditions encompass interdisciplinary issues that complement each other involving areas such as psychology, neuroscience, and psychiatry (6).

The WHO reports indicate that between 20 and 25% of adults suffer from a mental disorder at some point in their lives (7). Among the most reported conditions are anxiety disorders, depressive disorders, schizophrenia, personality disorders, childhood and adolescent disorders, disorders resulting from substance abuse, and other psychoses (8, 9). Physical illnesses, gender, age, conflicts, catastrophes, and family and social environments are factors that influence the prevalence of these disorders (8–10). In Brazil, epidemiological surveys carried out by the Ministry of Health pointed to a prevalence of 20% in the adult population, with 3% of the general population suffering from severe and persistent mental disorders, 12% needing some mental healthcare, whether continuous or occasional, and 6% having serious psychiatric disorders resulting from the use of alcohol and other drugs (10). Only 2.3% of the Brazilian National Health Service (NHS - SUS) annual budget is allocated to mental health (10).

Despite advances in restructuring the model, some issues remain major challenges for the Brazilian mental healthcare network. Epidemiological reports highlight the scarcity and discrepant distribution of services with underfunding, weakness in intra- and intersectoral alignment, the stigma related to psychological distress, and the difficulties in resocializing aggravated patients (11–13). In addition to reductions in funding and the delay in implementing new services, the inclusion of public mental health actions and policies that are not based on scientific evidence or recognized by international protocols has resulted in the incorporation of specialized outpatient clinics and psychiatric hospitals in the psychosocial care network (RAPS), thereby increasing the costs of psychiatric hospitalizations and the maintenance of therapeutic communities. This new reality points to a risk of shutting down public mental health services (14). This situation calls for the development of studies that collaborate with the debate about the articulation between different points of care, as the availability and scarcity of services have a negative impact on mental healthcare (11, 15, 16).

We highlight the hypothesis that the scarcity and distribution of these services impact intra- and intersectoral alignment, affecting patients in psychological distress, especially those who are female and in vulnerable situations, and worsening levels of anxiety and depression in these cases. This hypothesis has at its base the idea that improving the identification of networks between demands, regions, and clinical and epidemiological characteristics is a fundamental step in the development of public policies and good practices in mental health (15, 16). To date, no clinical or sociodemographic characterization of mental health has been conducted in the northwest region of São Paulo State, and thus, there are no accessible reports of these data. This study aimed to provide a description of the clinical and epidemiological profile of patients who use mental health services in this region. This is a necessity for all countries in order to establish protocols to promote improvements in the implementation of public mental health services and care for patients suffering from psychological distress (3, 13).

## 2 Participants and methods

### 2.1 Study location and population

**Figure 1:**
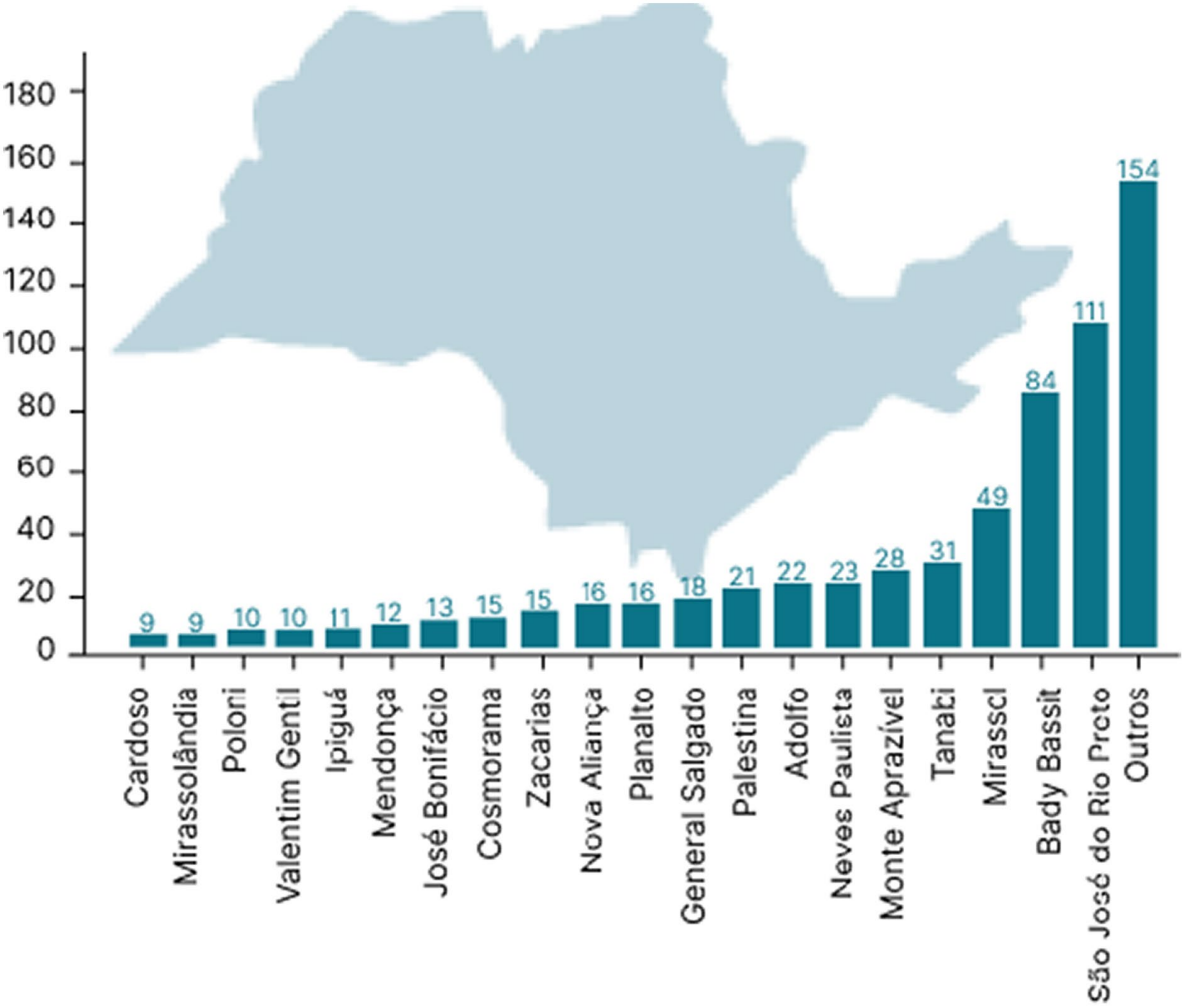
Map of the region covered by the psychiatric outpatient clinic of Hospital de Base HB-FUNFARME, São José do Rio Preto metropolitan region, São Paulo State, Brazil, and the distribution of patients’ origins attended. Map of the region of São José do Rio Preto adapted from https://pt.wikipedia.org/wiki/S%C3%A3o_Jos%C3%A9_do_Rio_Preto.

A retrospective descriptive observational study was performed with a quantitative approach. The survey was carried out at the Psychiatry Outpatient Clinic of Hospital de Base, located in the municipality of São José do Rio Preto, São Paulo, Brazil. As a public psychiatric outpatient clinic that receives psychiatric patients from the northwest of São Paulo State, it is possible to draw a specific picture of the region. The clinic offers multidisciplinary treatment for people with psychotic disorders, depression, anxiety, post-traumatic stress disorder, mood disorders, schizophrenia, and sexuality problems and for drug users, and child psychiatry care, in addition to carrying out general screening to refer patients for other therapies. The services offered are part of a treatment reference center of the Brazilian NHS (SUS) with patients being referred for diagnosis, monitoring, and mental health treatment. The patients come from a region that comprises 102 municipalities with more than 2 million inhabitants under the administration of the Regional Health Department XV (DRS XV) based in São José do Rio Preto, São Paulo state, Brazil (Figure 1).

The electronic medical records of patients who consulted in the psychiatry outpatient clinic of a teaching hospital, Hospital de Base, São José do Rio Preto, SP, Brazil, between March 2018 and March 2020 were reviewed. Data on psychiatric-related problems as itemized in the International Classification of Diseases—10th Edition (ICD-10), number of appointments, and prescribed treatments were collected. All patients with psychiatric disorders and with at least one appointment in the study period were included. Eight aspects were investigated: 1—disorder classification; 2—patient gender; 3—patient age; 4—patient origin; 5—symptoms at the last appointment; 6—number of medications prescribed at the last appointment; 7—illness duration; and 8—treatment duration. Patients who did not have any consultation for psychiatric care within the study period were excluded. These variables were chosen to characterize the main components listed in the MVPEP^®^ program of electronic medical records used by the Specialty Outpatient Clinic. This study was approved by the Research Ethics Committee of the Medical School of São José do Rio Preto (FAMERP) under registration number 4.040.192. Patients’ identities have all been kept confidential. Due to the retrospective nature of the study with data being collected from the hospital’s electronic medical records, patients enrolled in this study were not required to sign an informed consent form.

### 2.2 Data analysis

The data were organized in a spreadsheet and examined using Microsoft Office Excel 2010® software. The data were analyzed using descriptive statistics including absolute and relative frequencies and means and standard deviations. In addition, the chi-square and Fisher’s exact tests were used for comparisons using GraphPad Instant Software (Version 3.06). Multivariate logistic regression, used to verify the main diagnoses found and related treatment, was performed with the SPSS Program (IBM, version 23). Student’s *t*-test was used to evaluate differences in gender related to disease duration, whereas analysis of variance (ANOVA) was used to assess differences in the mean duration of the different types of disorders and the frequency of worsening of symptoms.

## 3 Results

A previous survey evaluated 3,861 patients, and thus, a sample size of 820 was estimated. Of 820 patients, 143 were excluded as they were treated by other medical departments and did not have an appointment at the psychiatry outpatient clinic within the study period. Patients from other regions of the country were also excluded from this sample. Hence, a total of 677 patients were included in this analysis using an estimated sampling error of 4.5% with a confidence level of 99%. In order to improve the accuracy of the inclusion criteria, only patients who received care at the psychiatric outpatient clinic during the study period were included. Patients treated by other medical services (hospital ward, intensive care unit, and emergency) who were not consulted at this psychiatric outpatient clinic during the study period and patients from other regions of the country were excluded. This filtering was necessary as the Hospital de Base in São José do Rio Preto, São Paulo, Brazil, is a regional center and provides many different types of services, such as emergency care, intensive care units, and outpatient clinics. Therefore, patients with psychiatric comorbidities who were entered into the hospital’s computer system were not necessarily attended by the outpatient psychiatric service but may have been evaluated for other types of psychiatric services (e.g., psychiatric consultations) or other services not linked to the Department of Psychiatry (i.e., clinical emergency and/or intensive care units).

A total of 8,384 appointments were identified in the study period. According to the ICD-10, the disorders registered were as follows: 1—generalized anxiety; 2—severe depressive episode without psychotic symptoms; 3—moderate depressive episode; 4—paranoid schizophrenia; 5—unspecified non-organic psychosis; 6—bipolar affective disorder; 7—recurrent depressive disorder and current episode of severe depression without psychotic symptoms; 8—unspecified anxiety disorder; 9—panic disorder (episodic paroxysmal anxiety); 10—organic delusional disorder (schizophrenic type); 11—recurrent depressive disorder; 12—mixed anxiety–depressive disorder; 13—mental and behavioral disorders due to alcohol use—dependence syndrome; and 14—mental and behavioral disorders due to the use of multiple drugs and the use of other psychoactive substances—dependence syndrome (Table 1).

**Table 1:** Epidemiological profile of appointments at the psychiatric outpatient clinic of Hospital de Base HB-FUNFARME (DRS XV), according to the ICD-10 codes, metropolitan region of São José do Rio Preto, São Paulo State, Brazil.

Category	Classification	n	(%)
F.062	Organic Delusional Disorder	159	1.90
F.102	Mental and Behavioral Disorders Due to Alcohol Use—Dependence Syndrome	386	4.60
F.192	Mental and Behavioral Disorders Due to Multiple Drug Use and Other Psychoactive Substance Use—Dependence Syndrome	227	2.71
F.200	Paranoid Schizophrenia	644	7.68
F.29	Non-Organic Psychosis Not Specified	189	2.25
F.310	Bipolar Affective Disorder, Hypomanic Current Episode	92	1.10
F.311	Bipolar Affective Disorder, Current Manic Episode Without Psychotic Symptoms	25	0.30
F.312	Bipolar Affective Disorder, Current Manic Episode With Psychotic Symptoms	23	0.27
F.314	Bipolar Affective Disorder, Major Depressive Current Episode Without Psychotic Symptoms	30	0.36
F.319	Unspecified Bipolar Affective Disorder	60	0.72
F.321	Moderate Depressive Episode	988	11.78
F.322	Severe Depressive Episode Without Psychotic Symptoms	194	2.31
F.331	Recurrent Depressive Disorder, Moderate Current Episode	853	10.17
F.332	Recurrent Depressive Disorder, Severe Current Episode Without Psychotic Symptoms	206	2.46
F.410	Panic Disorder [Episodic Paroxysmal Anxiety]	351	4.19
F.411	Generalized Anxiety	2,264	27.0
F.412	Mixed Anxiety and Depressive Disorder	1,254	14.96
F.419	Anxiety Disorder Not Specified	439	5.24

**Figure 2:**
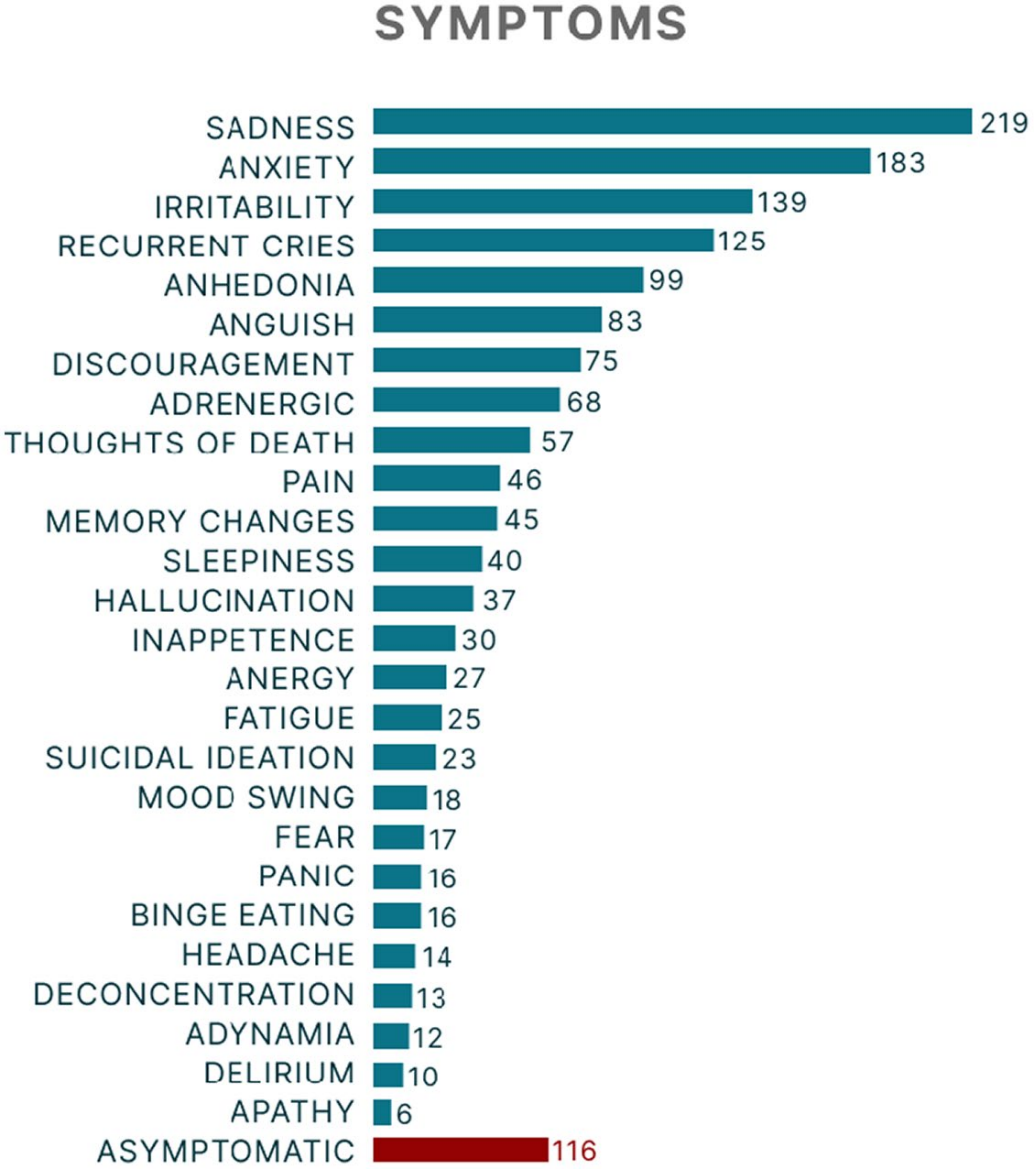
Prevalence of symptoms in patients who attended the psychiatric outpatient clinic of Hospital de Base HB-FUNFARME (DRS XV), a teaching hospital in São Paulo State, Brazil.

**Figure 3:**
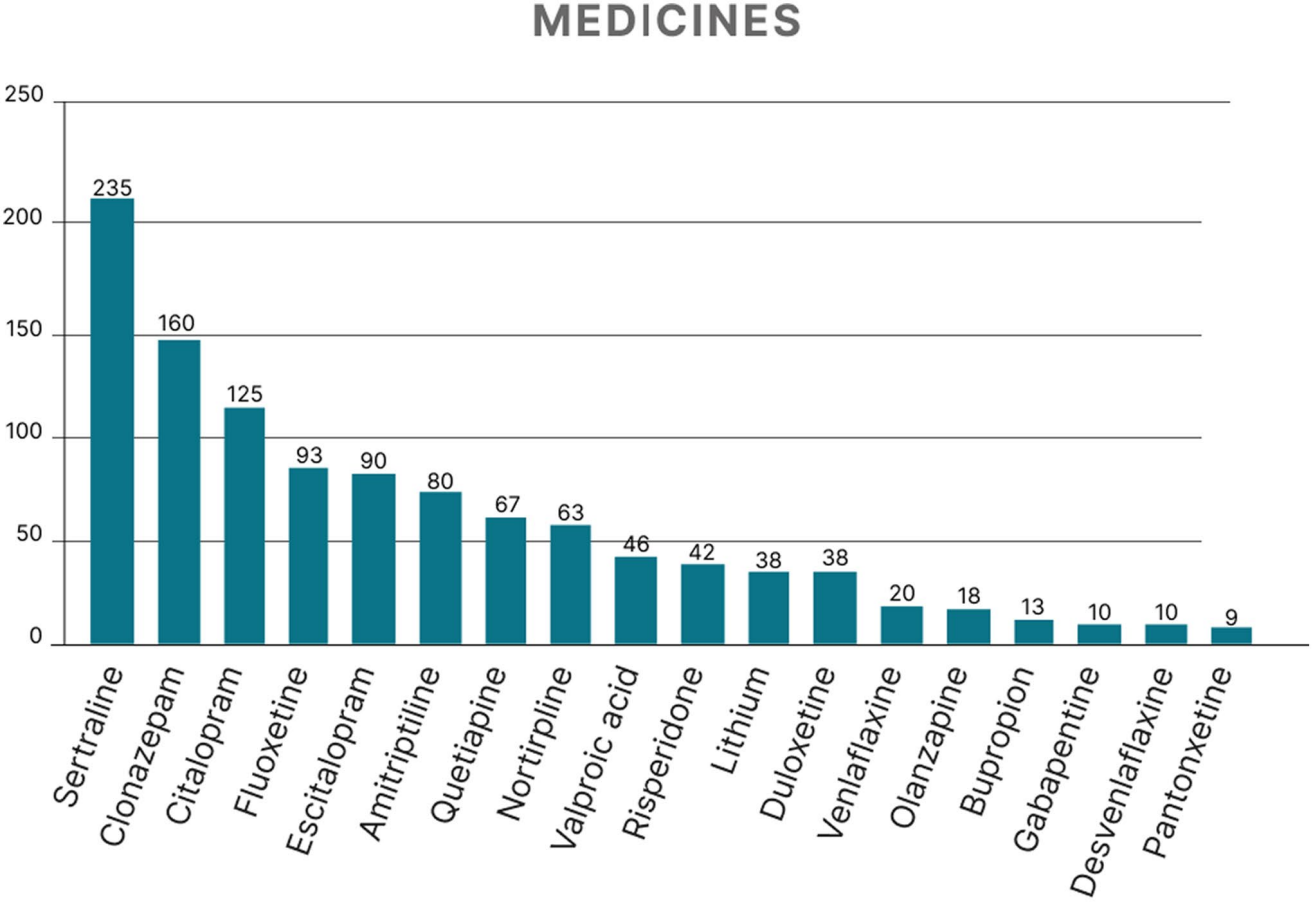
Medications prescribed to patients who attended the psychiatric outpatient clinic of a teaching hospital in São José do Rio Preto, São Paulo State, Brazil.

The most prevalent disorder was generalized anxiety disorder (GAD), followed by mixed anxiety–depressive disorder and moderate depressive episode (Table 1). Most patients (75.92%; *n* = 514) were female, and the mean age was 53.69 ± 11.65 years. During the patient’s last appointment, the most prevalent manifestations were anxiety and depressive symptoms such as sadness, anxiety, and irritability, although 17.13% of the patients were asymptomatic (Figure 2). The mean number of medications prescribed per patient at the last visit was 2.44 ± 1.44 with the highest means being for patients with bipolar affective disorder (BAD), current manic episode with psychotic symptoms (4.0 ± 1.41), and organic delusional disorder (3.84 ± 1.89). The most common medications prescribed were of the selective serotonin reuptake inhibitors class (SSRI), such as sertraline hydrochloride (Figure 3).

**Figure 4:**
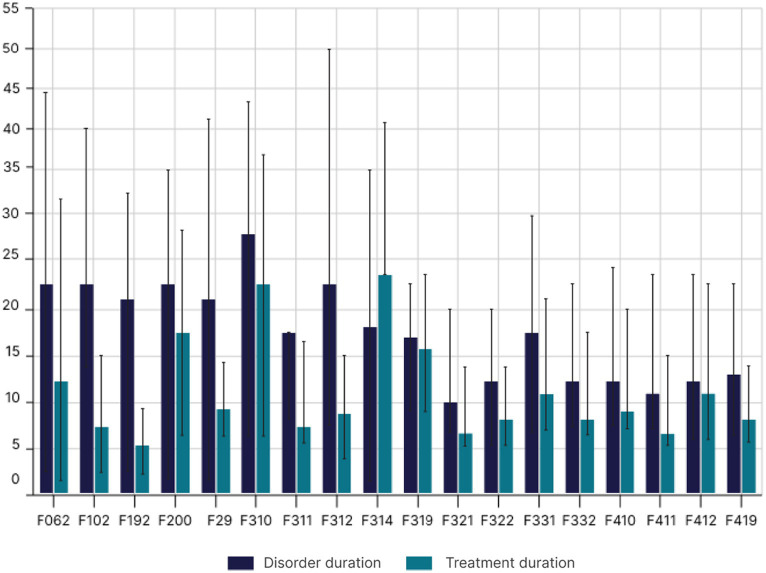
Treatment duration in years according to the disorder categorized using the ICD-10 codes.

**Figure 5:**
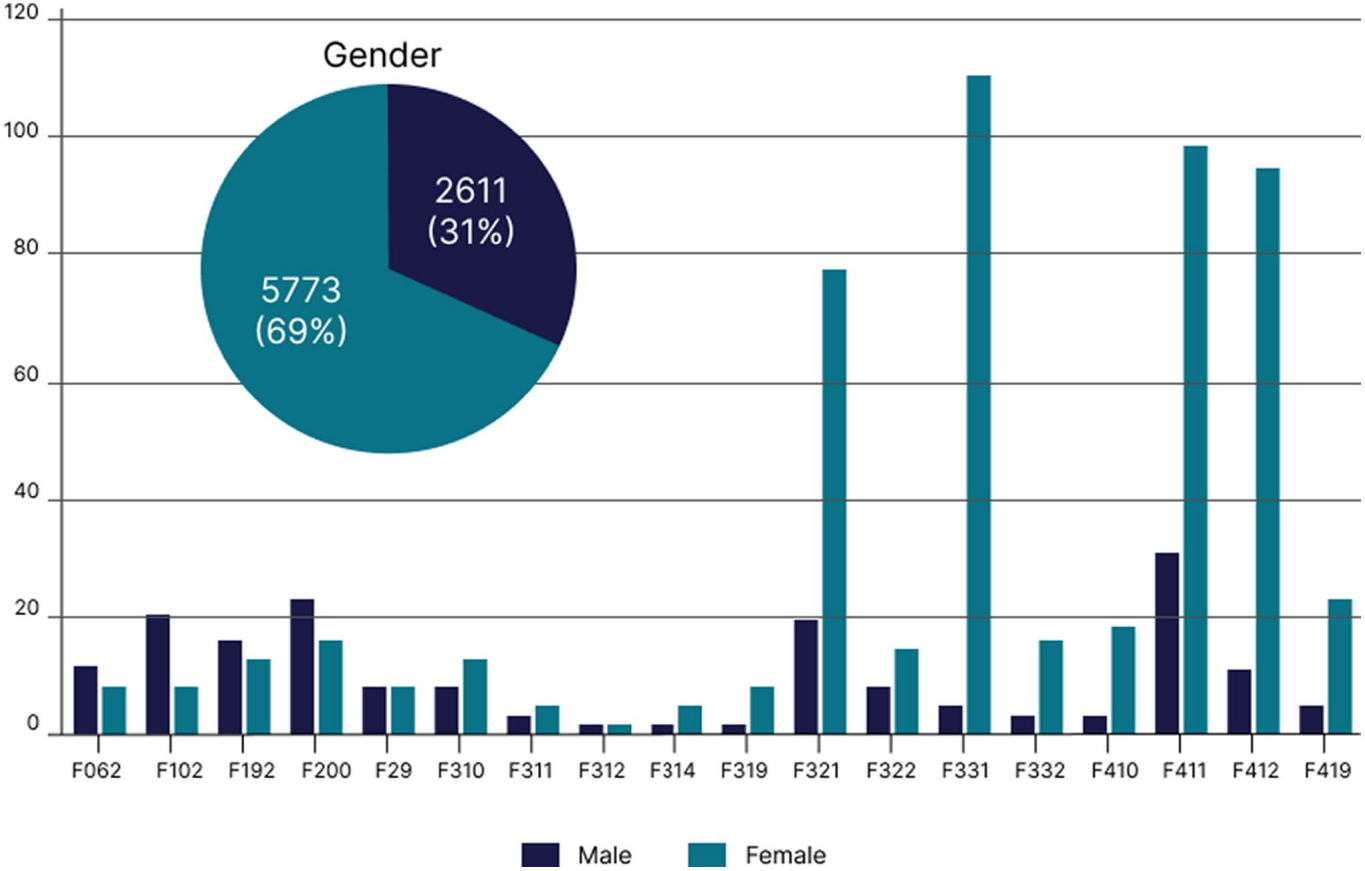
Distribution of disorders, according to ICD-10, by gender in patients who attended the psychiatric outpatient clinic of a teaching hospital, São Paulo State, Brazil.

The mean disease duration and treatment duration were 15.29 ± 13.32 years and 9.469 ± 10.11 years, respectively. Women had a mean disease duration of 14.28 ± 12.40 years, while for men it was 18.38 ± 15.47 years. The mean treatment duration for women was 9.65 ± 10.40 years, and for men, it was 8.87 ± 9.14 years. The diagnosis that had the longest mean duration of illness was bipolar affective disorder (current hypomanic episode), with a mean of 28.91 years (Figure 4). Moderate depressive episodes had the shortest mean duration of 9.96 years. There was a predominance of women (78.86%) with anxiety and depressive disorders in the generalized anxiety group. However, schizophrenia was predominant in men (59%) as were delusional disorder (57.89%), alcohol abuse (70%), and illicit substance abuse (60%; Figure 5). Disease duration correlated with gender is shown in Table 2.

**Table 2:** Correlation of gender with disease duration using the ICD-10 codes.

ICD-10 CODE	Female	Male	*p* value*
F.062	14 ± 11	11.2 ± 10	0.009
F.102	13.4 ± 9.2	7.4 ± 4.1	0.42
F.192	9 ± 7.8	5.4 ± 3.8	0.17
F.200	13 ± 10.4	11 ± 7.3	0.46
F.29	13.1 ± 9.0	15 ± 3.6	0.9
F.310	8.1 ± 4.7	11.9 ± 6.8	0.92
F.311	11 ± 6.1	7.4 ± 4.1	0.7
F.312	6.9 ± 2.7	5.1 ± 3.6	0.54
F.314	17.1 ± 12	11.2 ± 9.0	0.29
F.319	14 ± 6.1	12 ± 10.1	0.93
F.321	12 ± 7.8	9.2 ± 5.9	0.8
F.322	11.2 ± 7.5	8.2 ± 5.8	0.43
F.331	10.7 ± 5.4	9.7 ± 6.1	0.78
F.332	7.1 ± 4.3	8.2 ± 4.8	0.52
F.410	9.3 ± 6.5	10.2 ± 6.9	0.18
F.411	11.3 ± 5.7	8.1 ± 4.8	0.88
F.412	12.8 ± 4.0	10.3 ± 6.1	0.6
F.419	12.7 ± 8.1	9.4 ± 7.2	0.77

^*^Student’s t-test for independent samples.

Regarding the distribution of diagnoses, the chi-square test was used to assess the absence of equal distribution for each pair of diagnostic categories with three comparisons having significance: generalized anxiety, mixed anxiety and depressive disorder, and moderate depressive episode. The frequencies of generalized anxiety (ICD-10: F41.1; value of *p* = 0.015; OR: 2.441; 95%CI: 1.75–11.39), mixed anxiety and depressive disorder (ICD-10: F41.2; value of *p* = 0.013; OR: 2.743; 95%CI: 1.92–7.16), and moderate depressive episode (ICD-10: F32.1; value of *p* = 0.009; OR: – 2.137; 95%CI: 1.74–6.39) were significantly higher than other diagnoses (value of *p* < 0.0001 for each diagnostic category). The Nagelkerke r^2^ value was 0.050. For comparisons regarding drug treatment, the most frequent diagnosis identified in the previous comparisons was used (generalized anxiety ICD-10: F41.1) and included the three drugs as a dependent variable. There were statistically significant differences for the use of sertraline (value of *p* = 0.008; OR: 2.63; 95% CI: 2.62–12.43), clonazepam (value of *p* = 0.007; OR: 2.869; 95% CI: 1.11–8.14), and citalopram (value of *p* = 0.017; OR: 2.536; 95% CI: 1.85–7.34) compared to other medications, and a greater frequency of worsening of symptoms was observed in patients with moderate depressive episode (11.78%; *n* = 988) and mixed anxiety and depressive disorder (14.96%; *n* = 1,254; Table 3).

**Table 3:** Treatment duration of patients diagnosed with ICD-10 codes F.411, F.412, F.321, and the recommended drug treatments.

ICD-10	F41.1	F41.2	F32.1
Treatment duration (years)	7.21	9.62	4.32
Recommended drug treatment			
	Sertraline	Clonazepam	Citalopram
*p* value	0.008	0.007	0.017
OR	0.263	2.869	2.536
95% CI	2.62–12.43	1.11–8.14	1.85–7.34

OR, Odds ratio; CI, confidence interval.

As the psychiatric diagnoses observed presented differences in mean duration, we tested the possibility that our sample would present significant differences regarding the worsening of symptoms for the most frequent types of disorders observed. Comparisons were made using analysis of variance (ANOVA) with the results indicating that the group of patients with mixed anxiety and depressive disorder presented a statistically significant difference compared with moderate depressive episodes (value of *p* < 0.0001; Table 3), but the difference for the group with generalized anxiety disorder was not significant (value of *p* > 0.05).

## 4 Discussion

### 4.1 Mental disorder predictors in Brazil

Epidemiological surveys carried out by the Health Ministry have found a 20% prevalence of mental disorders in the adult population: 3% of the general population suffer from severe and persistent mental disorders, 12% need some ongoing or occasional mental healthcare, and 6% have severe psychiatric disorders resulting from the use of alcohol and other drugs. Currently, only 2.3% of the Brazilian NHS annual budget is allocated to Mental Health (8). The incidence of these disorders in public health service users was reported in a recent multicenter study involving four Brazilian state capitals. The rate was above 50%; it was especially high among women, the unemployed, and people with little education and low income (9–11). These results place Brazil as one of the countries with the highest number of anxious people in the world, demonstrating a high prevalence of mental disorders in the Brazilian population (12, 13). Refining the profile of these patients is detrimental to the promotion of more effective public mental healthcare policies and treatment. Furthermore, as a critical factor, research has shown the presence of common mental disorders (states of psychological distress, anxiety, depression, or somatoform symptoms that formally do not meet sufficient criteria for diagnoses of depression and/or anxiety according to the current classifications) manifesting together, increasing psychiatric comorbidity (13–15). These analyses highlight women with an average age of 53 years who require continuous or occasional mental healthcare associated with depression and anxiety as a predictor of greater vulnerability to mental disorders. However, the male gender was associated with more serious disorders such as schizophrenia as well as delusional disorder, alcohol abuse, and abuse of illicit substances.

### 4.2 Models of outpatient mental healthcare in the state of São Paulo

There is a paucity of studies and reference services in the field of mental health in the northwest region of São Paulo State. Detailed assessments are required regarding the regions covered by the service and the profile of the user served. Santos et al. (17) demonstrated that the outpatient model adopted by Dr. Jandira Masur Psychiatric Specialties Medical Outpatient Clinic in Vila Maria, São Paulo, is a reference in the management of mental health in the Brazilian NHS (17). The patients treated at this center are mostly women (62%), and the prevalent diagnoses are mood and anxiety disorders (36.5%), followed by stress-related disorders (29%) (17, 18). The findings in the current study demonstrate some clinical characteristics similar to the profile presented by this center. This indicates that coordination between the entire network of continued healthcare and the outpatient model can offer a greater comprehensiveness of mental healthcare (17–19).

The discussion about outpatient mental healthcare has recently taken other paths. The work carried out in these lines of action does not receive the necessary attention within the scope of public policies with a lack of knowledge regarding this model being demonstrated (19, 20). The Brazilian Psychiatry Association proposes the creation of services that more specifically meet the needs of the Brazilian population. The results of this study also suggest the necessity of the insertion of specialized outpatient clinics within the same debate as Psychosocial Care Centers (CAPS), the Family Health Strategy (ESF), psychiatric beds in general, and matrix support hospitals in Brazil (19–21). The fact that many disorders presented by the patients in the current sample are characterized by a chronic evolution, multidrug treatment and instability in the manifestation of symptoms, and treatment adherence indicates that many cases require continuous care due to more serious and persistent disorders, while less serious disorders require sporadic care. The reorganization of a network of services replacing the psychiatric hospital often includes a role for outpatient clinics (22–25).

There was a predominance of women with anxiety and depressive disorders with 78.86% of patients in the group of generalized anxiety being women. However, many patients present multiple conditions—anxiety and depression, or schizophrenia and bipolar disorder, for example, and there seems to be a tendency for these patients to present more than one diagnosis. Periods of control and remission of symptoms in these profiles are related to effective psychiatric monitoring associated with psychotherapy, psychoeducation programs, family therapy, home visits, occupational therapy, skill development, monitored medication, rehabilitation programs, self-help groups, and therapeutic monitoring, among others (25–29). The provision of specific services for realities like these contributes by improving the quality of mental healthcare and can dialog with the clinical perspective (27–29).

Clinical and epidemiological research has reformulated the work in these outpatient clinics based on a clinical perspective (30). One of the main changes was the inclusion of psychologists (30, 31). The majority of these professionals concentrated on health programs for HIV, hypertension, smoking, and diabetes, among others (30). It has been recommended that any suffering patient, regardless of their diagnosis, symptoms, age, gender, or even psychopathology, should have access to an expanded clinic and should be welcomed within the scope of mental health (30–32). This was substantial progress and resulted in the arrival of many untreated or poorly treated patients, whose psychological impairment had serious consequences on their daily lives (30). The impact of this practice reinforced the need for an increase in hiring human resources and indicated gaps in the way psychiatric treatment was being offered in outpatient clinics (with its focus on the clinical characteristics of pharmacotherapy) (30–33).

Studies on these practices have focused on the outpatient context (33). Regular meetings were established with teams, encouraging collaboration and communication among mental health professionals, and continually working together to receive clients in the form of the reception group (33, 34). The group of professionals was extended beyond psychiatrists and psychologists, with nurses, social workers, and occupational therapists being available in certain units (34, 35).

Santos et al. (17) highlight the importance of discussions in mental health forums from this standpoint (17, 18). They defined the mental health outpatient clinic as a legitimate clinical device in the care network, not only in cases considered mild or moderate but also in severe cases (17, 18, 34). This resizing represents a milestone as it serves as the basis for the new guidelines as reported in the document entitled *Recommendations on Mental HealthCare in the Basic Network* (36).

Data from the World Health Organization (WHO) indicate that during the years of the COVID-19 pandemic 93% of countries around the world have experienced some type of outage in mental health services (37). During this period, outpatient clinics specialized in mental health used information from international and national health authorities to make decisions to adapt standards and routines in units, providing data on the clinical profile of patients treated before and after the start of the pandemic (17, 38, 39). Furthermore, modifications described based on management reports, procedure manuals, minutes of meetings, and training presentations made it possible to maintain outpatient care during this period (17). The findings of the current study indicate similar characteristics. Of the consultations analyzed here, 65.2% (*n* = 5,466) took place via telemedicine and online assessments. In fact, the data collection period for the present sample was also affected by the COVID-19 pandemic as it included 1 year of lockdown. Different prevalences and factors emerged in this context. Global literature indicates that the epidemiological distribution of mental disorders and associated factors was heterogeneous in the general public, COVID-19 patients, and healthcare professionals (38, 40–42). The advantages of telemedicine in this situation are well documented; however, this modality may require greater skill of psychologists and psychiatrists, and more dialog between these and other professionals, as assessments are essentially clinical and may depend on interactionist factors at the time of evaluation (42, 43). Furthermore, evidence suggests that a psychiatric epidemic is occurring as a consequence of the pandemic and that clusters of symptoms such as those found in the current sample may be due to this situation (40, 41). Arafat et al. reported that situations of this order in telemedicine care used in the treatment of critical mental health issues still need to be precisely explored, especially with regard to diagnosis and its association with clinical symptomatological and sociodemographic characteristics (42).

These studies have demonstrated that there is a gap between cases with a very long duration of illness and treatment and newly diagnosed and treated cases with the same prevalent symptoms (sadness, anxiety, and irritability) (44). Similar to international reports (45, 46), this study also identified the predominance of anxiety and depressive disorders in women: 78.86% of the group with generalized anxiety were women. However, many patients had multiple conditions—anxiety and depression, for example, making them more likely to have more than one diagnosis. These data reinforce the need to implement a new mental healthcare model in Brazil, especially after the COVID-19 pandemic, based on the installation of a mental healthcare model guided by the psychosocial paradigm, which is directed toward the clients in their various dimensions within a socio-community context (47, 48). The RAPS proposal foresees the development of intersectoral actions and encourages the active participation of users who were unable to access mental health services that were not provided by CAPS, or who were not contemplated for comprehensive care beds or residential therapeutic services (SRT) (16, 49, 50). It also provides for the development of innovative care practices with the active participation of users (16, 49, 50).

### 4.3 RAPS and shared responsibility

Epidemiological reports on mental health from the last decade played a significant role in the creation of RAPS as an organizational proposal for mental health services in Brazil (49). The clinical profile and sociodemographic characteristics highlight the shared and interdisciplinary accountability of cases as an element capable of influencing hierarchical, pyramidal, and fragmented patterns and promoting continuous flows of care in appropriate technological spaces (13, 51). As a result, there was a reversal of spending on mental health with financial stimulation for substitute services, the closure of psychiatric beds in monovalent hospitals, and the expansion of community services (49).

The challenge for improving this model is to identify which populations are most in need and strengthen intersectoral coordination (13, 52, 53). Diagnostic practices need to establish mechanisms that encompass symptom profiles that are widely shared across populations and disorders (with regard to psychopathology), but others are more specific to one or a set of forms of psychopathology (53–55). The severity of symptoms and treatment time in certain regions may indicate the need for specific services for certain locations (54–56). As an example, the current sample indicated that diagnosis and treatment time are related, thereby reinforcing the need for a biopsychosocial model. The findings of this study revealed an average treatment time of 9.65 ± 10.40 years for women and 8.87 ± 9.14 years for men. The most common disorder that also had the longest treatment time was BAD (current hypomanic episode); with a mean of 28.91 years, it is associated with symptoms such as sadness, anxiety, and irritability (Figure 4). The moderate depressive episode at 9.96 years persisted for the shortest time. These characteristics represent more difficult sectoral demands as they are directly related to international nosologies (53–57).

There is a discussion about the need to improve the diagnosis (57, 58). The search for biomarkers has received increasing attention from the scientific community as it may help to establish more effective public policies in mental health (58). Research is moving toward the idea that perhaps there is a general dimensionality of predisposition in the psychic and biological patterns of disorders (58–61). However, these results still need to be officially organized.

**Figure 6:**
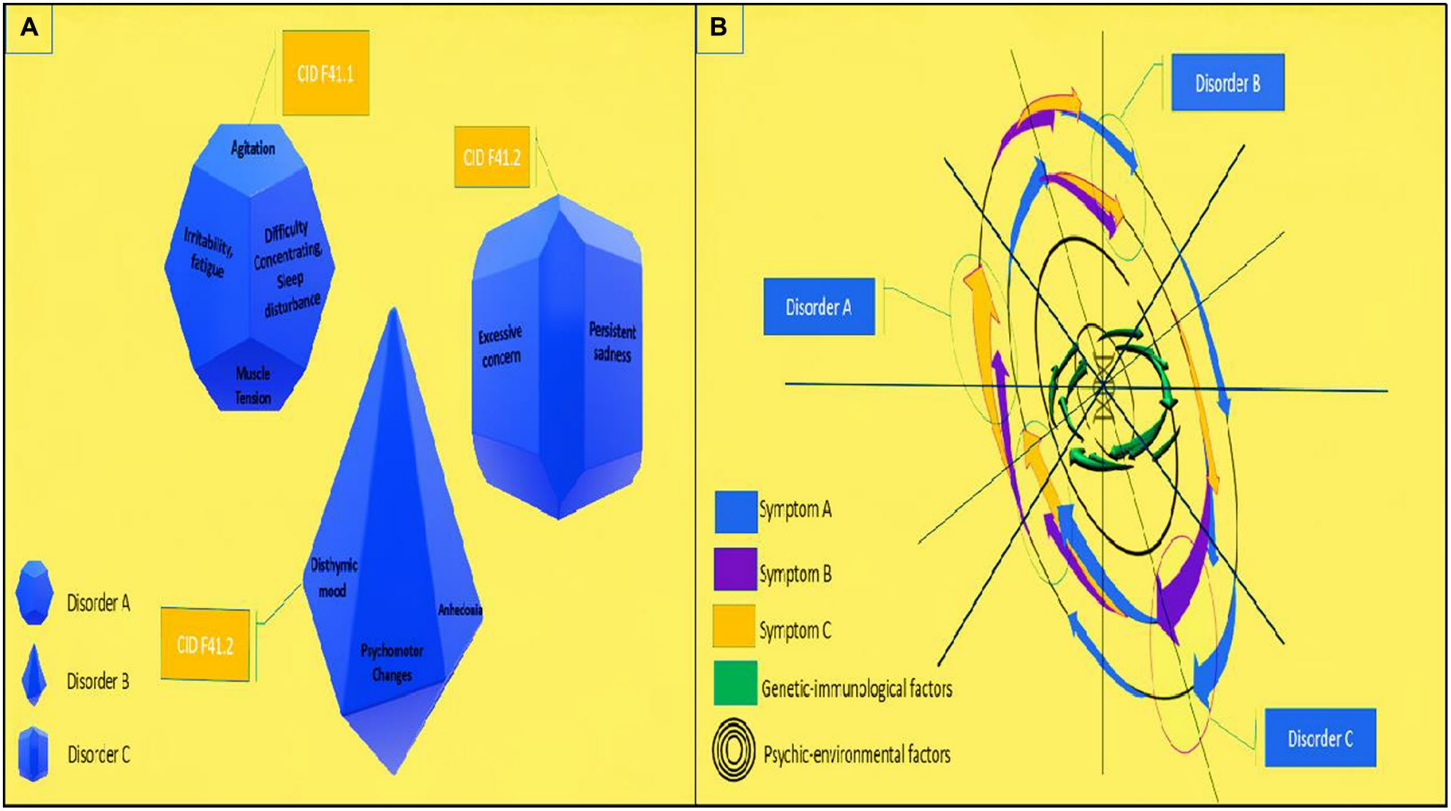
Proposal for transdiagnostic evaluation in mental health based on continuous variables for mental disorders: **(A)** categorical variables for mental disorders (current); **(B)** proposal for evaluation on continuous variables for mental disorders.

The challenge for the assumption of dimensionality is to identify what the main reference dimensions are and quantify them with respect to the population. In this model, two patients who present the same symptoms, but different sets of mutations or psychoneuroimmunological patterns, could be diagnosed and treated differently (Figure 6). It is also necessary to verify whether a single dimension is capable of measuring psychopathology, its comorbidity, the severity of symptoms, and its response to treatment over time (59, 60). In practice, the community perspective implies the development of this scale across a wide range of services, contexts, and locations. This process, which has not yet begun in many regions and countries, aims to ensure that some of the functions of mental institutions are fully provided and that stereotypes of patients in psychological distress are not perpetuated (61–76).

### 4.4 Limitations of the study

As a descriptive retrospective observational study carried out using electronic medical records, this research identified small data errors, with some incomplete and/or missing information. This is an important factor and can increase the difficulty in analysis. Some records did not present a detailed longitudinal analysis of patients, such as the presence or absence of adverse events during treatment, higher levels of social vulnerability, and greater history of mental disorders in the family and cultural variables. These factors may represent common precedents for the etiology and development of disorders and serve as a basis for new epidemiological analysis in mental health (69–76).

## 5 Conclusion

Anxiety and depressive disorders were the most prevalent MHDs in a Brazilian public tertiary psychiatric outpatient clinic. Additionally, patients were mostly female, and the ages ranged from 30 to 60 years old. A high coexistence of symptoms, as observed in other regions, was also observed here. Most disorders proved to share anxiety and depressive symptoms, especially GAD and depressive disorder; disease duration, on average, was approximately 15 years and treatment approximately 10 years. SSRIs were the most prescribed medications, with at least one medication prescribed at the last visit. These data provide evidence for the necessity of establishing more specific protocols in mental health and advocating the dimensional transdiagnostic approach as an aid to public mental health services.

Further analyses are needed in order to dimensionally assess common background predictors (higher rate of negative histories during development, higher levels of social vulnerability, and greater history of mental disorders in the family) and genetic and immunological traits in different disorders. This same basis of elements in the etiology of disorders demands a biopsychosocial assessment and a dimensional transdiagnostic evaluation of mental health.

## Data availability statement

The original contributions presented in the study are included in the article/supplementary material, further inquiries can be directed to the corresponding authors.

## Ethics statement

The studies involving humans were approved by Research Ethics Committee of Medical School of São José do Rio Preto—FAMERP, under registration number 4.040.192 (CAAE 28371320.7.0000.5415). The studies were conducted in accordance with the local legislation and institutional requirements. The ethics committee/institutional review board waived the requirement of written informed consent for participation from the participants or the participants’ legal guardians/next of kin because We have performed a retrospective-descriptive study using only data from the eletronic outpatient appointments from the Psychiatry Outpatient Clinics.

## Author contributions

GC: Conceptualization, Data curation, Formal analysis, Investigation, Methodology, Validation, Writing – original draft. MG: Data curation, Formal analysis, Investigation, Methodology, Writing – original draft. GA: Data curation, Funding acquisition, Investigation, Resources, Supervision, Validation, Visualization, Writing – review & editing. CA: Data curation, Formal analysis, Investigation, Methodology, Writing – original draft, Writing – review & editing. AL: Data curation, Investigation, Methodology, Writing – original draft. FB: Data curation, Investigation, Methodology, Supervision, Validation, Writing – review & editing. LM: Data curation, Resources, Validation, Writing – review & editing. CB: Conceptualization, Funding acquisition, Investigation, Project administration, Supervision, Writing – review & editing.
